# Lymph Node Dissection Morbidity in Thyroid Cancer: An Integrative Review

**DOI:** 10.14744/SEMB.2021.33401

**Published:** 2021-12-29

**Authors:** Antonella Pino, Carmelo Mazzeo, Francesco Frattini, Daqi Zhang, Che-Wei Wu, Guido Zanghì, Gianlorenzo Dionigi

**Affiliations:** 1. Division of General Surgery, Policlinic G. Martino University of Messina, Messina, Italy; 2. Division of General Surgery, ASST Settelaghi, Varese, Italy; 3. Division of Thyroid Surgery, China-Japan Union Hospital of Jilin University, Jilin Provincial Key Laboratory of Surgical Translational Medicine, Jilin Provincial Precision Medicine Laboratory of Molecular Biology and Translational Medicine on Differentiated Thyroid Carcinoma, Changchun, People; 4. Department of Otolaryngology-Head and Neck Surgery, Kaohsiung Medical University Hospital, Kaohsiung Medical University, Kaohsiung, Taiwan; Faculty of Medicine, College of Medicine, Kaohsiung Medical University, Kaohsiung, Taiwan; 5. Department of General Surgery and Medical-Surgical Specialties, Policlinico - Vittorio Emanuele Hospital, University of Catania, Italy; 6. Division of General Surgery, Endocrine Surgery Section, Istituto Auxologico Italiano IRCCS, Milan, Italy; Department of Pathophysiology and Transplantation, University of Milan, Italy

**Keywords:** Nerve lesions, hypoparathyroidism, secondary bleeding, pleural injury, esophageal injury

## Abstract

Cervical lymphadenectomy is a common procedure for thyroid cancer. Some of the complications are congruent with the complications of thyroid surgery, in particular recurrent laryngeal nerve paresis and hypoparathyroidism as well as bleeding and wound infection. Specific complications of lateral cervical lymph node dissection are injuries to the accessory, phrenic and hypoglossal nerves, and the cervical plexus trunk and injuries, the salivary glands, and the lymphatic system, especially the ductus thoracicus. Most of these complications are very rare with an incidence of <1%. Profound anatomical knowledge and a careful dissection technique make a decisive contribution to minimizing complications.

Since the first description of cervical lymph node dissection over 100 years ago, the operation has been a standardized method for the treatment of metastases from tumors of the head-and-neck area.^[1,2]^ Operations on the thyroid and parathyroid glands, in particular, account for the largest share of all operations in the neck area.^[3]^ Operations are often performed in connection with malignant tumors of the thyroid and parathyroid glands.^[3,4]^ In this respect, the complication management of these operations is of particular importance. Serious intraoperative complications that require immediate correction and control are comparatively rare. In this respect, avoiding them plays a central role.

A distinction must be made between the structures of the central compartment in the so-called central lymphadenectomy with the structures of the recurrent laryngeal nerve (RLN), parathyroid gland, thyroid vessels, esophagus and trachea, and the lateral compartment (classification according to Robbins^[5]^), which contains the vascular nerve sheath with the vagus nerve, the common carotid artery and the internal jugular vein, the neural structures of the lateral compartment, and the lymphatic system. If the location is high in the cervical region, there is also a risk of injury from the parotid or submandibular gland.

Recurrent interventions in particular, but also previous radiation treatments, can present the surgeon with great challenges due to scarring, with the resulting increased complication rates.

## RLN

**Figure 1.**
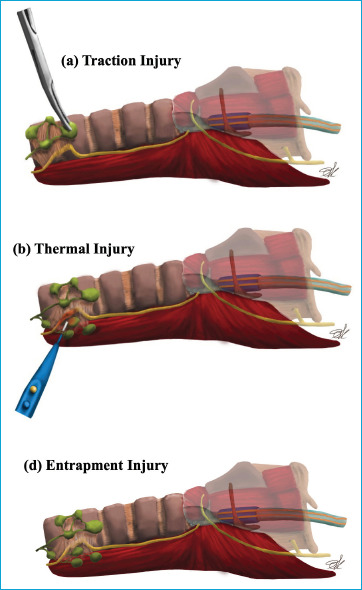
Mechanism of RLN injury while central lymph node dissection.

Since thyroid and parathyroid surgeries with possibly accompanying central lymphadenectomy are by far the most common interventions in the head-and-neck area, the identification and protection of the RLN and the superior laryngeal nerve (SLN) play a major role. Knowledge of the correct anatomy, the course and the position variations is essential (Fig. 1).

For lateral lymph node dissection, it is important to know the position of the accessory nerve, the hypoglossal nerve, the cervical plexus, the phrenic nerve, the vagus nerve and the minor occipital nerve.

## SLN

The SLN, which emerges from the vagus nerve like the RLN, branches off immediately below the inferior nervi vagi ganglion and runs medial to the internal carotid artery. As it progresses, it divides into a motorized external branch, which runs mediocaudally from the superior thyroid artery into the cricothyroid muscle, and a sensitive internal branch, which runs craniomedially to the superior thyroid artery and, after perforation of the thyrohyoid membrane, supplies the larynx mucosa.

Information on the frequency of damage to the external branch of the SLN (EBSLN) varies between 0 and 58% (Table 1).^[6]^ The partially high damage rates of the EBSLN are partly due to the variability of the anatomical course in relation to the position of the upper pole vessels. EBSLN appears to be particularly at risk on the left side (37.5% damage on the left and 12.5% on the right).^[7]^ The preparation near the capsule, especially the near-pole ligature, reduces the risk of the EBSLN lesion. However, routine exposure of the nerve is not recommended.

**Table 1.** Frequency of complications from cervical lymph node dissection

**Morbidity**	**Incidence (%)**
*N. phrenicus*	0.14
*N. vagus*	0.29
Recurrent laryngeal nerve	0ec.2 permanent
Superior laryngeal nerve	0upe
Plexus cervicalis	0.29
*N. accessorius*	1.34 ac
*N. hypoglossus*	0.29
Sympathetic trunk	?
Hypoparathyroidism	0is.4 permanent/8.7 pe temporary
Bleeding	0.29ee.7
Lymphatic fistula	0.5ym
Infection	0nfe.1
Tracheal injury	?
Pleura injury	?
Esophageal injury	0.14

## Cervical Plexus

The neck is mainly innervated by branches of the cervical plexus, which runs between the anterior and the median scalene muscles in the lateral neck triangle.^[8]^ The sensory fibers of the cervical plexus supply, through the nervus occipitalis minor, nervus auricularis magnus, nervus transversus colli and the nervus supraclaviculares, and the skin between the ear and below the clavicle. The motor fibers include the ansa cervicalis, the phrenic nerve, and the rami sternocleidomastoideus and trapezius and supply the prevertebral neck muscles, the scalene muscles, the diaphragm, and part of the trapezius and sternocleidomastoideus muscles.

### N. accessorius

The accessory nerve is one of the most frequently injured nerves in the lateral compartment, especially when lymph nodes are excised in the lateral neck triangle. The nerve runs through the sternocleidomastoid muscle to the trapezius muscle, which it supplies with motor fibers. In some cases, injury to the accessory nerve is not immediately apparent. In the post-operative check-up, the patients report painfully restricted mobility of the arm during abduction and rotation of the shoulder joint.^[9]^ Lesions of the nerve have a particularly good prognosis since their reconstruction is accompanied by good functional results even after more than 6 months.^[10]^

### N. hypoglossus

Injuries to the hypoglossal nerve are also rare occurrences. In addition to neck dissection, nerve injuries occur during operations on the carotid artery or as a rare symptom in carotid dissections.^[11]^ The hypoglossal nerve runs lateral to the internal and external carotid arteries as well as over the internal jugular vein and runs in an arch between the hyoid and hyoglossal muscles to the inner and outer muscles of the tongue.^[8,12]^ One-sided damage to the nerve leads to one-sided paralysis of the tongue with deviation to the diseased side. Difficulties in ingesting food and fluids and articulation disorders can occur. Damage on both sides leads to complete paralysis of the tongue, longer damage to atrophy of the tongue muscles.

## Sympathetic Trunk

The sympathetic trunk is the sympathetic pathway in the neck area, which runs right and left along the entire spine. The fibers from C8 (cervical vertebra 8) to T1 (thoracic vertebra 1) develop into the superior cervicalis ganglion, medium cervicalis ganglion, inferior cervicalis ganglion, and ganglion stellatum, this is the fusion of the ganglion cervicale inferius and the first thoracic ganglion.

Horner’s syndrome is a complex of symptoms caused by damage to the sympathetic nerve fibers of the ganglion stellatum. In the symptom triad, ptosis occurs due to the failure of the superior tarsal muscle, miosis occurs due to the failure of the dilatator muscle, both accompanied by anidrosis. In addition, the ptosis of the eyeball appears to have sunk (so-called pseudoenophthalmos).

## Parathyroid Glands

Hypoparathyroidism after thyroid surgery is reported in the literature to be up to 20%.^[13]^ The underlying disease and the extent of the resection have a clear influence on the risk of developing hypoparathyroidism postoperatively. Lymphadenectomy, in particular, increases the risk of devascularization because the inferior thyroid artery is often tied near the trunk.^[14-16]^

As part of the central, cervical lymphadenectomy, devascularization of the lower parathyroid glands (LPG) can occur in particular.

In cases of suspected underperfusion or accidentally removed LPGs, they are usually autotransplanted.^[17-20]^

## Bleeding

Bleeding after surgery on the neck is rather rare. However, they can be potentially life threatening in the case of compression and edema of the neck viscera. The incidence of post-operative bleeding is between 0 and 6.5%. In addition to taking anticoagulants and coagulation disorders, risk factors for bleeding are, above all, the extent of the resection, the higher age of the patient, the male sex, and the redo surgery. What the bleeding complications have in common is that they only occur later than 24 h after the end of the operation in rare exceptions.^[21,22]^

## Lymph Vessel Injury/ductus Thoracicus/Chylothorax

The rate of post-operative chyle fistulas due to injuries to the lymphatic system or the ductus thoracicus is around 1–8%.^[23–25]^ The lymph leakage occurs on the left side in three-quarters of the cases.

The ductus thoracicus runs in a curved course into the left vein angle, formed from the subclavian and internal jugular veins. Shortly before its confluence, this absorbs the lymph from the left arm, the left half of the head and neck, and the left half of the chest cavity.

Intraoperative treatment of the thoracic duct injuries, employing ligature or piercing ligature, appears sensible. Due to the thin wall of the vessel, sealing using electricity or an ultrasound dissector does not seem to be sufficient. Early surgical treatment is recommended if there is significant lymph leakage postoperatively (>300 ml/day). More minor leaks can often be healed with conservative therapy (drainage without suction, parenteral nutrition, or nutrition with medium chain triglycerides).^[26]^ In the case of extensive lymphadenectomy, the authors usually insert a drain to identify a possible lymph leakage. As a rare complication, a chylothorax can develop, resulting in effusion, dyspnea, or mediastinal displacement. In these cases, pleural ultrasonography and the installation of a drain seem to be necessary for diagnosis and therapy.^[27,28]^ The drainage shows a milky, cloudy puncture fluid with a high triglyceride content. A chyle leak can cause metabolic disorder through protein loss and a loss of immunoglobulins, vitamins, and electrolytes. Adequate fluid and electrolyte replacement should be ensured in the acute phase.

The benefits of octreotide in the healing of chyle fistulas have been extensively described in the literature. The final assessment, however, has not yet been adequately substantiated by a larger study.

## Wound Infection

With good wound management, infections rarely cause major difficulties. The infection rate is given as 0.2–14.1% (Table 1). Wound infections usually occur as a secondarily infected hematoma or in chyle fistulas. A significant and rare infection in lymph node dissection is mediastinitis.^[22]^ Hematogenous spread of the germs and a septic course rarely occur.

Antibiotic prophylaxis can be considered to avoid wound infections, especially in the case of extensive, time-consuming resection, and patient-specific risk factors.^[29-34]^

## Tracheal Injury

Intraoperative detection and early treatment of tracheal injuries have the best prognosis. Smaller lesions can be detected in a water bath with ventilation. Abscesses and mediastinitis increase the risk of suture insufficiency. The most feared complication is injury to the posterior wall of the trachea. Therefore, bronchoscopic monitoring is recommended in percutaneous tracheotomy. A conservative treatment of the injury with a stent or tube is possible, especially in the case of small lesions <2 cm and when prior irradiation has taken place and/or the cuff can be placed below the lesion. Small transmural injuries can be treated with a primary suture (monofilament and absorbable). As a safeguard, especially in the case of difficult lesions (posterior wall), the provision of additional material such as the pericardium, pleura, esophagus, or muscle with good blood supply (sternocleidomastoideus) is important. A peri- and post-operative antibiotic therapy is recommended in any case.

## Esophagus

Injuries to the esophagus involve extensive cervical tumor interventions in rare cases, in the exception of difficult goiter operations. As a rule, these are tangential injuries that can be closed with a direct suture. Antibiotic cover and, if necessary, drainage insert can prevent infection and abscess formation. In the case of larger defects and difficult sutures, the muscle flap can be useful. Extensive resections and plastic reconstructions are more likely to be reserved for secondary surgery.

## Pleural Injury/tension Pneumothorax

A deep cervical resection can result in a pleural injury in the area of the lung tips. These should be closed if possible; a leak test can be carried out using a water test. The possibility of intraoperative X-ray control should be given, so that in the rare case of a pneumothorax, a pulmonary drainage can be introduced intraoperatively.

## Salivary Gland/salivary Fistula

Injuries to the salivary glands with the resulting salivary fistula are extremely rare and only occur in 0.14% of cases in case series (Table 1).^[23]^ In the majority of cases of an injury with a subsequent salivary fistula, a conservative procedure causes the fistula to heal.

## Conclusion

The lymph node compartments of the neck are not clearly defined anatomical spaces comparable to the abdominal cavity or the chest. Lymph node dissection is based on landmarks such as blood vessels or muscles. In this respect, lymph node dissection consists in removing lymph and fat tissue within these defined anatomical landmarks and boundaries. Finally, a mention goes to the cases of reinterventions. The risk of injury to all the structures as mentioned above is considerably higher considering a subverted anatomy and the presence of adhesions as a result of the previous surgery. The most damaged structures are the trachea and the inferior laryngeal nerve followed by the esophagus and large vessels of the neck.

An anatomically exact knowledge and precise representation of the anatomical structures are, therefore, the basic principle for a low complication and at the same time oncologically radical surgery.

## Disclosures

**Peer-review:** Externally peer-reviewed.

**Conflict of Interest:** None declared.

**Authorship Contributions:** Concept – A.P., F.F.; Design – C.M.; Supervision – G.D.; Materials – A.P.; F.F.; Data collection &/or processing – D.Z.; Analysis and/or interpretation – D.Z.; C.W.W.; Literature search – G.Z.; Writing – A.P., G.D.; Critical review – C.W.W., G.D.

## References

[1] Crile G (1906). Excision of cancer of the head and neck.. JAMA.

[2] Ferrari CC, Rausei S, Amico F, Boni L, Chiang FY, Wu CW (2016). Recurrent laryngeal nerve injury in thyroid surgery: Clinical pathways and resources consumption.. Head Neck.

[3] Dionigi G, Bacuzzi A, Boni L, Rausei S, Rovera F, Dionigi R (2012). Visualization versus neuromonitoring of recurrent laryngeal nerves during thyroidectomy: what about the costs?. World J Surg.

[4] Kim EY, Eisele DW, Goldberg AN, Maselli J, Kezirian EJ (2011). Neck dissections in the United States from 2000 to 2006: volume, indications, and regionalization.. Head Neck.

[5] Robbins KT, Clayman G, Levine PA, Medina J, Sessions R, Shaha A, American Head and Neck Society; American Academy of Otolaryngology--Head and Neck Surgery (2002). Neck dissection classification update: revisions proposed by the American Head and Neck Society and the American Academy of Otolaryngology-Head and Neck Surgery.. Arch Otolaryngol Head Neck Surg.

[6] Aluffi P, Policarpo M, Cherovac C, Olina M, Dosdegani R, Pia F (2001). Post-thyroidectomy superior laryngeal nerve injury.. Eur Arch Otorhinolaryngol.

[7] Zhang D, Wang T, Kim HY, Wang P, Dionigi G, Pino A (2020). Strategies for superior thyroid pole dissection in transoral thyroidectomy: a video operative guide.. Surg Endosc.

[8] Zilles SS (1999). Anatomie..

[9] Zhang D, Pino A, Caruso E, Dionigi G, Sun H (2020). Neural monitoring in thyroid surgery is here to stay.. Gland Surg.

[10] Camp SJ, Birch R (2011). Injuries to the spinal accessory nerve: a lesson to surgeons.. J Bone Joint Surg Br.

[11] Jurkiewicz MT, Stein JM, Learned KO, Nasrallah IM, Loevner LA (2019). Hypoglossal nerve palsy due to carotid artery dissection: an uncommon presentation of a common problem.. Neuroradiol J.

[12] Kariuki BN, Butt F, Mandela P, Odula P (2018). Surgical anatomy of the cervical part of the hypoglossal nerve.. Craniomaxillofac Trauma Reconstr.

[13] Lombardi D, Accorona R, Paderno A, Cappelli C, Nicolai P (2017). Morbidity of central neck dissection for papillary thyroid cancer.. Gland Surg.

[14] Dralle H, Lorenz K, Machens A (2011). State of the art: surgery for endemic goiter--a plea for individualizing the extent of resection instead of heading for routine total thyroidectomy.. Langenbecks Arch Surg.

[15] Järhult J, Andersson PO, Duncker L (2012). Alternating from subtotal thyroid resection to total thyroidectomy in the treatment of Graves' disease prevents recurrences but increases the frequency of permanent hypoparathyroidism.. Langenbecks Arch Surg.

[16] Thomusch O, Machens A, Sekulla C, Ukkat J, Brauckhoff M, Dralle H (2003). The impact of surgical technique on postoperative hypoparathyroidism in bilateral thyroid surgery: a multivariate analysis of 5846 consecutive patients.. Surgery.

[17] Smith MA, Jarosz H, Hessel P, Lawrence AM, Paloyan E (1990). Parathyroid autotransplantation in total thyroidectomy.. Am Surg.

[18] Shaha AR, Burnett C, Jaffe BM (1991). Parathyroid autotransplantation during thyroid surgery.. J Surg Oncol.

[19] Zhang D, Wang T, Dionigi G, Zhang J, Zhao Y, Xue G (2019). Comparison of parathyroid hormone kinetics in endoscopic thyroidectomy via bilateral areola with open thyroidectomy.. BMC Surg.

[20] Zhang D, Wang T, Dionigi G, Fu Y, Zhang J, Zhao Y (2019). Application of carbon nanoparticles in endoscopic thyroidectomy via bilateral areola approach: total thyroidectomy plus central lymph node dissection.. J Laparoendosc Adv Surg Tech A.

[21] Zhang D, Zhang J, Dionigi G, Li F, Wang T, Li H (2019). Recurrent laryngeal nerve morbidity: lessons from endoscopic via bilateral areola and open thyroidectomy technique.. World J Surg.

[22] Zhang D, Fu Y, Zhou L, Liang N, Wang T, Del Rio P (2021). Thyroid surgery during coronavirus-19 pandemic phases I, II and III: lessons learned in China, South Korea, Iran and Italy.. J Endocrinol Invest.

[23] Polistena A, Monacelli M, Lucchini R, Triola R, Conti C, Avenia S, eet al (2015). Surgical morbidity of cervical lymphadenectomy for thyroid cancer: A retrospective cohort study over 25 years.. Int J Surg.

[24] Sakai A, Okami K, Onuki J, Miyasaka M, Furuya H, Iida M (2008). Statistical analysis of post-operative complications after head and neck surgery.. Tokai J Exp Clin Med.

[25] Duque CS, Sánchez JG, Dionigi G (2017). Chyle fistula in advanced and metastatic thyroid cancer.. Gland Surg.

[26] Lorenz K, Abuazab M, Sekulla C, Nguyen-Thanh P, Brauckhoff M, Dralle H (2010). Management of lymph fistulas in thyroid surgery.. Langenbecks Arch Surg.

[27] Sharma AK, Sahli ZT, Mathur A (2018). Bilateral chylothorax following reoperative central neck dissection for metastatic papillary thyroid cancer.. BMJ Case Rep.

[28] Ierardi AM, Pappalardo V, Liu X, Wu CW, Anuwong A, Kim HY (2016). Usefulness of CBCT and guidance software for percutaneous embolization of a lymphatic leakage after thyroidectomy for cancer.. Gland Surg.

[29] Fachinetti A, Chiappa C, Arlant V, Kim HY, Liu X, Sun H (2017). Antibiotic prophylaxis in thyroid surgery.. Gland Surg.

[30] Dionigi G, Boni L, Rovera F, Rausei S, Dionigi R (2011). Wound morbidity in mini-invasive thyroidectomy.. Surg Endosc.

[31] Dionigi G, Rovera F, Boni L, Dionigi R (2008). Surveillance of surgical site infections after thyroidectomy in a one-day surgery setting.. Int J Surg.

[32] Dionigi R, Dionigi G, Rovera F, Boni L (2006). Postoperative fever.. Surg Infect (Larchmt).

[33] Dionigi G, Rovera F, Boni L, Castano P, Dionigi R (2006). Surgical site infections after thyroidectomy.. Surg Infect (Larchmt).

[34] Dionigi R, Rovera F, Dionigi G, Imperatori A, Ferrari A, Dionigi P (2001). Risk factors in surgery.. J Chemother.

